# Radiation knowledge and anxiety levels among residents proximity to the world’s first AP1000 nuclear power unit

**DOI:** 10.3389/fpubh.2024.1532278

**Published:** 2025-01-14

**Authors:** Jiadi Guo, Zhiqiang Xuan, Cuiping Lei, Taotao Zheng, Zhongjun Lai, Xiaoji Hao, Shunfei Yu, Yiyao Cao

**Affiliations:** ^1^Department of Occupational Health and Radiological Protection, Zhejiang Provincial Center for Disease Control and Prevention, Hangzhou, Zhejiang, China; ^2^National Institute for Radiological Protection, Chinese Center for Disease Control and Prevention, Beijing, China; ^3^Section of Environmental Health, Sanmen County Center for Disease Control and Prevention, Taizhou, Zhejiang, China

**Keywords:** radiation-related knowledge, nuclear energy-related knowledge, nuclear power plant, anxiety, public acceptance

## Abstract

**Objective:**

Assess the level of radiation-related knowledge (RRK) and nuclear energy-related knowledge (NERK) among residents near the Sanmen Nuclear Power Plant, the first project adopted the Advanced Passive Pressurized Water Reactor (AP1000) technology.

**Methods:**

In this study, respondents were selected using stratified multi-stage random sampling for residents aged 18 years and above living within 30 kilometers of the Sanmen Nuclear Power Station. Respondents were surveyed face-to-face by investigators who received standardized training. The results of the survey were collated and analysed to assess the RRK and NERK levels of the respondents from both subjective and objective perspectives, and the anxiety levels were assessed using the Likert Scale. Factors affecting RRK, NERK and anxiety levels of residents were analysed using multiple linear regression analysis.

**Results:**

The study interviewed 751 individuals. Participants correctly answered an average of 2.76 out of 7 objective radiation knowledge questions, yielding a 39.4% RRK cognition rate. For nuclear energy knowledge, the average was 2.14 out of 7, resulting in a 30.5% NERK cognition rate. Spearman’s correlation and multiple linear regression analyses revealed that higher education and younger age were positively correlated with RRK and NERK. Gender significantly influenced NERK, with males scoring higher than females. Anxiety levels were inversely related to age and directly related to education. Regression analyses also indicated that occupation affected nuclear-related anxiety, and married and unmarried individuals exhibited higher anxiety levels than widowed individuals.

**Conclusion:**

Residents near the Sanmen Nuclear Power Station showed improvements in RRK and NERK, but levels remained low. Both RRK and NERK correlated with age and education, while NERK was also linked to gender. Anxiety among residents was associated with age, education, occupation, and marital status. These findings highlight the need for improved public education on RRK and NERK, effective engagement strategies, and measures to address residents’ anxiety to enhance decision-making and social trust regarding nuclear safety.

## 1 Introduction

With the escalating challenges posed by global climate change, it is urgent to achieve a clean and low-carbon transformation of the energy system. The Paris Agreement reached by the global climate change negotiations in 2015 established the long-term goal of “limiting the global average temperature rise by the end of this century to no more than 2°C compared to the pre-industrial revolution level, and working towards 1.5°C.” Energy production currently contributes more than half of global greenhouse gas emissions, and “decarbonization” has become the main direction of emission reduction (1). According to the annual report of the International Atomic Energy Agency (IAEA), nuclear power stands out for having the lowest greenhouse gas emissions per unit during its life cycle compared to other energy sources (2). In 2021, nuclear power supplied approximately 2653.1 TWh of greenhouse gas-free electricity, constituting around 10% of worldwide electricity generation and over a quarter of global low-carbon electricity production (3). In the same year, nuclear power generation in the United States accounted for 19.6% of the country’s total electricity generation; China’s nuclear power generation was 407.5 TWh, the second largest in the world after the United States, but nuclear power generation accounted for only 5.0% of the country’s national electricity generation (4). Therefore, with the goal of carbon peaking and carbon neutrality, China’s energy system will continue to accelerate the clean and low-carbon transition and actively develop nuclear power in a safe and orderly way (1).

While nuclear energy undergoes global development, the potential risks associated with it cannot be ignored. Major accidents like Chernobyl and Fukushima have heightened safety concerns at national and societal levels. Balancing public apprehension with the need for reliable, affordable electricity significantly influences political decisions. Despite advancements in waste technology and safety standards, public unease has rendered nuclear energy contentious. Public sentiment now plays a pivotal role in determining the fate of nuclear power initiatives (5).

The public’s perception of nuclear power risk is influenced by their knowledge, impacting attitudes toward nuclear plant construction (6–8). Following the Fukushima accident, Huang Lei et al. studied Chinese residents near the Tianwan plant, finding increased risk perception and decreased acceptance of nuclear power (9). Subsequent surveys on Asian university students revealed Chinese and Japanese students leaning towards phasing out nuclear plants compared to South Korean and Taiwanese peers (10). He Yanmin et al. analyzed safety perceptions among Chinese, Japanese, and Korean residents, showing that 40.5% of Chinese respondents indicated that they were in favor of it, the highest percentage among the three countries, followed by 16.1% in South Korea and 8.1% in Japan (11). It is not difficult to find that the public is anxious about nuclear power based on the results of the above study on nuclear power safety awareness and perception survey, and the occurrence of nuclear power plant accidents has aggravated this uneasiness. Despite persisting anxiety post-accidents, recent research by scholars like Wu (12) and Guo (13) suggested a fading of such concerns over a decade post-Fukushima, necessitating reevaluation.

Starting at Qinshan in Zhejiang in 1991, China’s nuclear power industry has thrived. Zhejiang currently houses one-fifth of the country’s operational units and contributes 16% to the national capacity. The Sanmen Nuclear Power Project, which is the first project adopted the world’s most advanced third-generation Advanced Passive Pressurized Water Reactor (AP1000) technology, is located in Sanmen County, on the eastern coastal area of Zhejiang Province. This study focused on assessing the level of radiation-related knowledge (RRK) and nuclear energy-related knowledge (NERK) among residents near the Sanmen Nuclear Power Plant in Zhejiang, as well as investigating residents’ acceptance of nuclear power. Through face-to-face surveys, the research aimed to uncover factors influencing public acceptance of nuclear power, providing valuable insights for promoting nuclear development in China.

## 2 Methods

### 2.1 Study subjects

The study involved participants over 18 years living within 30 km of the Sanmen Nuclear Power Plant. The population was divided into two groups: the general public, encompassing “Commercial and service workers,” “Agriculture, forestry, animal husbandry, fishery, and water conservancy workers,” “Machine operators and production transport workers,” “Miscellaneous employed individuals,” and “Students”; and the representative group, which included “Government and party officials, corporate and public institution leaders,” “Professional technicians,” and “Clerks and related workers.”

### 2.2 Sampling method

This survey aimed to estimate the awareness rates(%) of RRK and NERK among residents near the nuclear power plant and compare rates across different populations. The sample size was determined using the formula for estimating the overall rate *p* for simple random sampling. The calculation formula was:


$$n=\frac{z_{a}^{2}\times pq}{d^{2}}$$

According to the 2009 Nuclear and Radiation Awareness Survey of residents near the Qinshan Nuclear Power Plant, the radiation awareness rate was 39.6% (14). For sample size calculations, the awareness rate was set at *p* = 0.4, with a permissible error of *d* = 0.1 × *p*. For *α* = 0.05, *z* = 1.96, and *n* was 576 residents. Considering stratified sampling with a design effect *deff* < 1 and accounting for a 10% non-response rate, the total sample size was adjusted to 634.

Stratified multi-stage random sampling was employed, using the No. 1 reactor of the nuclear power plant as the center. The surrounding area was divided into three concentric strata: 0–10 km, 10–20 km, and 20–30 km. Two villages were randomly selected from each stratum, with 120 residents surveyed per village, resulting in 240 individuals per stratum and a total of 720 participants. The proportion of opinion-representative individuals in each stratum was capped at 25% (60 individuals). When significant economic, cultural, or dietary differences existed within the survey area, these factors were considered in the stratified sampling, prioritizing diversity. Additionally, efforts were made to ensure that the gender and age distribution of respondents reflected that of the local population.

### 2.3 Questionnaire

The questionnaire survey was conducted through face-to-face interviews by trained investigators with medical backgrounds from provincial, county-level CDCs and local health centers. They received standardized training from experts at the Chinese Centre for Disease Control and Prevention (China CDC). Respondents completed the questionnaires based on clear explanations of the survey’s purpose and content provided by the surveyors. If respondents encountered difficulties due to cultural limitations or other reasons, the surveyor would clarify the questions and record the responses accordingly.

The questionnaire was based on the “Questionnaire on Nuclear Radiation Knowledge of Residents around Nuclear Power Plants” prepared by the National Institute for Radiological Protection of China CDC, which has been utilized in various surveys assessing residents’ RRK and NERK around nuclear power plants. The questionnaire consisted of two parts: Part 1 gathered residents’ basic personal information, including gender, age, residence, marital status, and occupation. Part 2 assessed RRK and NERK through questions about residents’ perceptions, evaluations of nuclear power plant safety, and attitudes toward nuclear energy development.

The questionnaire on residents’ RRK included eight questions, divided into two parts. The first part contained a subjective evaluation question that assessed respondents’ self-perceived knowledge of radiation with five options ranging from “very little” to “very knowledgeable.” The second part consisted of seven objective questions, designed to assess factual knowledge about radiation. The questions were as follows: (1) “Natural radiation does not exist in the environment where we live?” (Correct answer: “wrong”); (2) “Are you not exposed to radiation when you fly in an airplane?” (Correct answer: “wrong”); (3) “When you have a CT scan in a hospital, you will not be exposed to radiation?” (Correct answer: “wrong”); (4) “Is it a sign of ionizing radiation (☢)?” (Correct answer: “yes”); (5) “Do radioactive substances all have the same half-life?” (Correct answer: “no”); (6) “Some of the spices we eat have been irradiated by artificial radiation?” (Correct answer: “yes”); (7)“Can mobile phones produce electromagnetic radiation?” (Correct answer: “yes”). Each correct response earned one point, and the results were summarized based on the respondents’ answers. Additionally, respondents were categorized into five groups according to their subjective evaluations, with analysis conducted on group size, proportion of total respondents, objective knowledge scores, and education levels.

The questionnaire on residents’ NERK comprised eight questions, divided into two parts. Part 1 assessed subjective perceptions, mirroring the format used in the evaluation of RRK. Part 2 featured seven objective questions designed to gauge factual knowledge about nuclear energy: (1) “Have serious accidents occurred at nuclear power plants in Hiroshima and Nagasaki, Japan?” (Correct answer: “wrong”); (2) “Did a serious nuclear power plant accident occur in Fukushima, Japan?” (Correct answer: “yes”); (3) “Is uranium the main component of nuclear fuel used in nuclear power plants?” (Correct answer: “yes”); (4) “Solid waste from nuclear power plants, including used nuclear fuel, is not radioactive?” (Correct answer: “no”); (5) “Once a nuclear power plant is shut down, does the reactor stop releasing heat immediately?” (Correct answer: “yes”); (6) “Do nuclear power plants, like coal-fired power plants, require a continuous supply of fuel to generate electricity?” (Correct answer: “yes”); (7) “Is the type of nuclear reaction in China’s commercial nuclear power plants nuclear fission?” (Correct answer: “yes”). The assessment of NERK employed the identical methodology used for RRK.

The residents’ anxiety levels were evaluated using a Likert scale, which incorporated responses to four queries: (1) “What is your perception of the nuclear power plant’s safety?” with options ranging from “very safe” to “very unsafe”; (2) “Do you have concerns regarding a potential serious accident at the plant?” with options from “not at all worried” to “very worried”; (3) “How likely do you perceive a terrorist attack on the nuclear power plant?” with options from “very unlikely” to “very likely”; (4) “Are you concerned about the health risks associated with the nuclear power plant?” with options from “not at all concerned” to “very concerned.” Responses were scored on a scale from 1 to 5, with “1” representing the most positive response and “5″ the most negative. Responses of “I do not know” were not scored. The scores from the four questions were then summed to calculate the final level of anxiety.

### 2.4 Quality control

Before the survey, all investigators underwent standardized training. Completed questionnaires were signed by the investigators and scrutinized by a verifier for completeness, legibility, and logical soundness. Any discrepancies prompted follow-up with respondents for correction, with the verifier then signing for validation. Daily debriefings were conducted to address survey issues and quality control, facilitating the continuous refinement of procedures. Data from the questionnaires were dually entered and cross-checked using the EpiData 3.1 software.

### 2.5 Statistical analysis

Statistical analysis was conducted using SPSS 22.0 software. Continuous data that did not meet the normality test were described using the median (*M*) and the 25th and 75th percentiles [*M*(*P*25, *P*75)]. The Mann–Whitney *U* test was used for comparisons between two groups, and the Kruskal-Wallis test was used for comparisons between multiple groups. The relationship between variables and influencing factors was assessed using Spearman correlation analysis. Multiple linear regression analysis was employed to analyze the factors affecting the RRK, NERK, and anxiety levels of residents living near the nuclear power plant. A difference was considered statistically significant at *p* < 0.05. The RRK awareness was determined by the ratio of correctly answered questions to the total, expressed as a percentage, and the NERK awareness was calculated using an identical method.

## 3 Results

### 3.1 Demographic characteristics of the study participants

In accordance with the sample size calculation formula, the minimum required sample size for this study was 634 individuals. The final survey encompassed 751 individuals, exceeding the requirement. The demographic breakdown revealed 590 members of the general public, 78.6% of the sample, with a median age (*P*25, *P*75) of 43.0 (33.0, 56.0) years. The remaining 161 were opinion representatives, 21.4% of the total, with an age of 35.0 (27.0, 43.0) years.

The study population was categorized by gender into 358 males (47.7%) and 393 females (52.3%), with median ages (*P*25, *P*75) of 43.0 (31.0, 55.0) years for males and 39.0 (30.0, 51.5) for females. The age disparity between genders was not statistically significant (*H* = 2.602, *p* = 0.107). Participants were also categorized into five age groups, with counts of 81, 196, 164, 152, and 158 for the age ranges 18 to 25, 26 to 35, 36 to 45, 46 to 55, and over 55, respectively.

Participants were stratified into eight occupational groups: “Government and party officials, corporate and public institution leaders,” “Professional technicians,” “Clerks and related workers,” “Commercial and service workers,” “Agriculture, forestry, animal husbandry, fishery, and water conservancy workers,” “Machine operators and production transport workers,” “Miscellaneous employed individuals,” and “Students.” The respective group sizes were 15, 122, 24, 100, 198, 130, 156, and 6.

Educational levels were categorized into six groups: “Illiterate,” “Primary school,” “Junior middle school,” “High school, technical school and technical secondary school,” “Junior college,” and “Undergraduate and above.” The corresponding participant counts were 66, 119, 230, 167, 77, and 92.

Participants were also divided into three groups based on the proximity of their residences to the nuclear power plant: 0–10 km (254 individuals), 10–20 km (254 individuals), and 20–30 km (243 individuals). The median ages were 40.0 (31.0, 53.3), 39.0 (29.0, 52.0), and 43.0 (31.0, 55.0) years, respectively, with no significant differences across groups (*H* = 1.695, *p* = 0.428).

Residential classification revealed: 102 urban respondents (13.6%) with an age of 34.0 (26.0, 41.0) years; and 649 rural respondents (86.4%) with an age of 43.0 (32.0, 55.0) years.

Respondents were categorized into four marital status groups: “Married,” “Divorced,” “Widowed,” and “Unmarried,” with group sizes of 603, 16, 14, and 118, respectively. Additionally, 6.8% (51 individuals) of respondents had family members employed at the nuclear power plant. For comprehensive demographic data, refer to Table 1.

**Table 1:** General characteristics of the study population (*n* = 751).

Variable	*n*	%	Age (years old) *M* (*P*25, *P*75)	*p* value
Population classification				<0.001
General public	590	78.6	43.0 (33.0, 56.0)	
Opinion representative	161	21.4	35.0 (27.0, 43.0)	
Sex				0.107
Male	358	47.7	43.0 (31.0, 55.0)	
Female	393	52.3	39.0 (30.0, 51.5)	
Age (years old)				<0.001
18–25	81	10.8	24.0 (22.0, 25.0)	
26–35	196	26.1	30.0 (28.0, 33.0)	
36–45	164	21.8	39.0 (38.0, 42.0)	
46–55	152	20.2	50.0 (48.0, 53.0)	
>55	158	21.1^a^	64.0 (59.0, 69.3)	
Occupation				<0.001
Government and party officials, corporate and public institution leaders	15	2.0	40.0 (26.0, 57.0)	
Professional technicians	122	16.2	33.0 (26.0, 43.0)	
Clerks and related workers	24	3.2	38.0 (29.5, 48.8)	
Commercial and service workers	100	13.3	39.0 (29.0, 49.0)	
Agriculture, forestry, animal husbandry, fishery, and water conservancy workers	198	26.4	55.0 (48.0, 64.0)	
Machine operators and production transport workers	130	17.3	32.5 (27.0, 38.0)	
Miscellaneous employed individuals	156	20.8	43.0 (34.0, 55.8)	
Students	6	0.8	19.0 (18.0, 19.0)	
Educational level				<0.001
Illiterate	66	8.8	63.0 (56.0, 69.0)	
Primary school	119	15.8	54.0 (49.0, 64.0)	
Junior middle school	230	30.6	44.0 (38.0, 52.0)	
High school, technical school and technical secondary school	167	22.2	32.0 (27.0, 40.0)	
Junior college	77	10.3	28.0 (24.0, 34.0)	
Undergraduate and above	92	12.3	33.0 (27.0, 38.8)	
Distance from nuclear power plant (km)				0.428
0–10	254	33.8	40.0 (31.0, 53.3)	
10–20	254	33.8	39.0 (29.0, 52.0)	
20–30	243	32.4	43.0 (31.0, 55.0)	
Location				<0.001
Town	102	13.6	34.0 (26.0, 41.0)	
Village	649	86.4	43.0 (32.0, 55.0)	
Family members work at nuclear power plants				0.338
No	700	93.2	40.0 (31.0, 53.0)	
Yes	51	6.8	36.0 (28.0, 53.0)	
Marital status				<0.001
Married	603	80.3	44.0 (35.0, 55.0)	
Divorced	16	2.1	46.0 (38.0, 53.8)	
Widowed	14	1.9	66.0 (53.5, 72.3)	
Unmarried	118	15.7	25.0 (22.0, 28.0)	

a When the total percentage does not equal 100% after rounding, redistribute the difference to the last group of data.

### 3.2 The awareness of RRK

The objective evaluation of RRK required an understanding of common concepts, which included recognizing the ubiquity of natural background radiation, gauging radiation exposure from air travel and CT scans, acknowledging the variability in radioactive material half-lives, and discerning the electromagnetic radiation emitted by mobile phones. It also involved assessing the reliability of signage for ionizing radiation.

#### 3.2.1 Subjective evaluation of RRK

Respondents evaluated their own RRK. A significant 18.9% (*n* = 142) admitted to being very ignorant, while 54.5% (*n* = 409) felt they were not very knowledgeable. Moderately knowledgeable individuals comprised 17.4% (*n* = 131), and 7.6% (*n* = 57) rated their knowledge as somewhat high. Interestingly, a small 1.6% (*n* = 12) considered themselves to be very knowledgeable about radiation.

#### 3.2.2 Objective evaluation of RRK

Survey results indicated that only 4.9% (*n* = 37) of respondents correctly answered all seven radiation knowledge questions, while 21.8% (*n* = 164) answered none correctly. The average number of correctly answered objective radiation knowledge questions was 2.76, with an overall RRK awareness rate of 39.4%. A Mann–Whitney U test revealed a statistically significant difference in RRK scores between opinion-represented individuals [5.0 (3.0, 6.0)] and the general public [2.0 (0.0, 4.0)], with *U* = 22622.0 and *p* < 0.001.

#### 3.2.3 Differences between subjective and objective ratings

Based on respondents’ self-assessment of their RRK, participants were categorized into five groups, with objective scores calculated for each. A positive correlation was observed as respondents with increasing self-assessed knowledge levels of “Ignorant,” “Not very knowledgeable,” and “Moderate” also had correspondingly higher objective scores, suggesting alignment between self-perception and measured knowledge. Concurrently, the educational level of these groups showed a rising trend. Conversely, for those who rated themselves as “quite knowledgeable” and “very knowledgeable,” a paradoxical decrease in both objective scores and educational levels was noted. The objective evaluation scores were found to correspond with the level of education. Detailed results of both subjective and objective evaluations are presented in Table 2.

**Table 2:** Differences between subjective and objective ratings of RRK.

Subjective evaluation	*n*	%	Objective valuation	Education level
Ignorant	142	18.9	1.0	2.0
Not very knowledgeable	409	54.5	3.0	3.0
Moderate	131	17.4	5.0	4.0
Quite knowledgeable	57	7.6	4.0	4.0
Very knowledgeable	12	1.6	3.0	3.0

### 3.3 The awareness of NERK

The questions objectively assessing NERK centered on nuclear power plant operations, including topics like accidents, fuel, waste management, and reactor dynamics.

#### 3.3.1 Subjective evaluation of NERK

Respondents were surveyed on their self-assessed knowledge of nuclear energy. A substantial 24.8% (*n* = 186) admitted to being very ignorant, while 58.9% (*n* = 442) felt they were not very knowledgeable. Moderately knowledgeable individuals comprised 12.0% (*n* = 90), and 3.5% (*n* = 26) rated their knowledge as somewhat high. In addition, a minimal 0.8% (*n* = 7) considered themselves very knowledgeable about nuclear energy.

#### 3.3.2 Objective evaluation of NERK

Survey results revealed that a mere 0.3% (*n* = 2) of respondents correctly answered all seven questions, while 31.7% (*n* = 238) answered none correctly. On average, respondents answered 2.14 questions correctly, yielding a NERK awareness rate of 30.5%. The Mann–Whitney U test showed that opinionated individuals had higher NERK awareness scores [4.0 (2.0, 5.0)] than the general public [1.0 (0.0, 3.0)], and the difference was statistically significant (*U* = 25199.5, *p* < 0.001).

#### 3.3.3 Differences between subjective and objective ratings

Respondents self-assessed their NERK, which categorized them into five groups with corresponding objective scores. As subjective ratings of knowledge increased from “Ignorant” to “Quite knowledgeable,” objective scores aligned with this trend, indicating a match between self-assessment and objective evaluation. The educational level of respondents in these four categories also correlated positively with their self-assessed knowledge. However, a discrepancy was noted in the “very knowledgeable” group, where educational attainment was unexpectedly lower than in the “quite knowledgeable” group. The objective scores were generally reflective of the level of education. Detailed results of both subjective and objective evaluations are presented in Table 3.

**Table 3:** Differences between subjective and objective ratings of NERK.

Subjective evaluation	*n*	%	Objective evaluation	Education level
Ignorant	186	24.8	0.0	2.0
Not very knowledgeable	442	58.9	2.0	3.0
Moderate	90	12.0	4.0	4.0
Quite knowledgeable	26	3.5	4.0	4.0
Very knowledgeable	7	0.8^a^	4.0	3.0

a When the total percentage does not equal 100% after rounding, redistribute the difference to the last group of data.

### 3.4 Factors affecting the cognition of RRK and NERK

#### 3.4.1 Population classification

**Figure 1:**
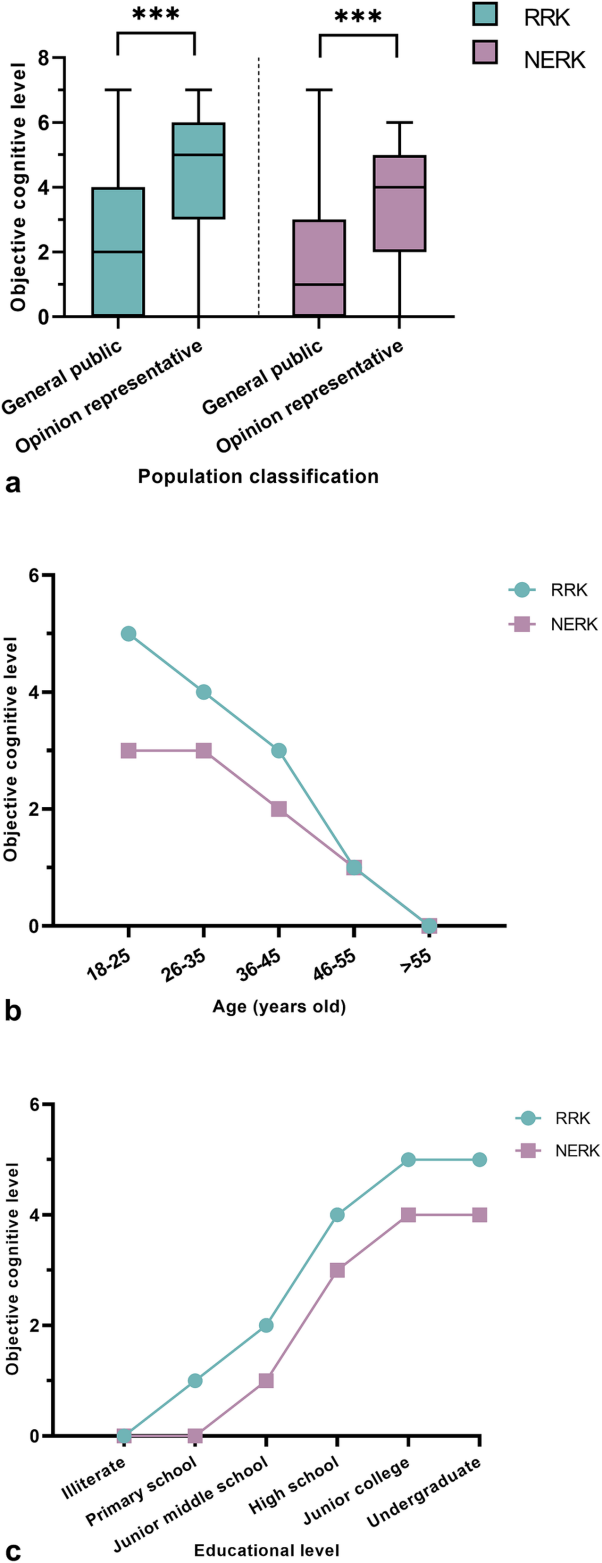
Factors influencing the cognition of RRK and NERK. **(A)** The influence of the population classification among the respondents on the cognition of RRK and NERK. The solid line represents the average cognitive expression level of the two groups. ***, *p* < 0.001; **(B)** the influence of the age classification among the respondents on the cognition of RRK and NERK; **(C)** the influence of the education classification among the respondents on the cognition of RRK and NERK.

The objective cognition of RRK and NERK was significantly higher in the group of opinion representatives (*n* = 161) than in the group of the general public (*n* = 590; *U* = 22622.0, *p* < 0.001; *U* = 25199.5, *p* < 0.001; Figure 1A).

#### 3.4.2 Age classification

Spearman correlation analysis showed that the level of objective cognition of RRK and NERK was negatively correlated with age (*r* = −0.526, *p* < 0.001; *r* = −0.415, *p* < 0.001), indicating that the level of objective cognition of RRK and NERK decreased with the increase in the age of the respondents (Figure 1B).

#### 3.4.3 Education level

Conversely, Spearman correlation analysis showed that the level of objective perception of RRK and NERK was positively correlated with the respondents’ education level (*r* = 0.664, *p* < 0.001; *r* = 0.598, *p* < 0.001), indicating that the level of objective perception of RRK and NERK increased with the increase of the respondents’ education level (Figure 1C).

### 3.5 Factors influencing respondents’ anxiety levels

**Figure 2:**
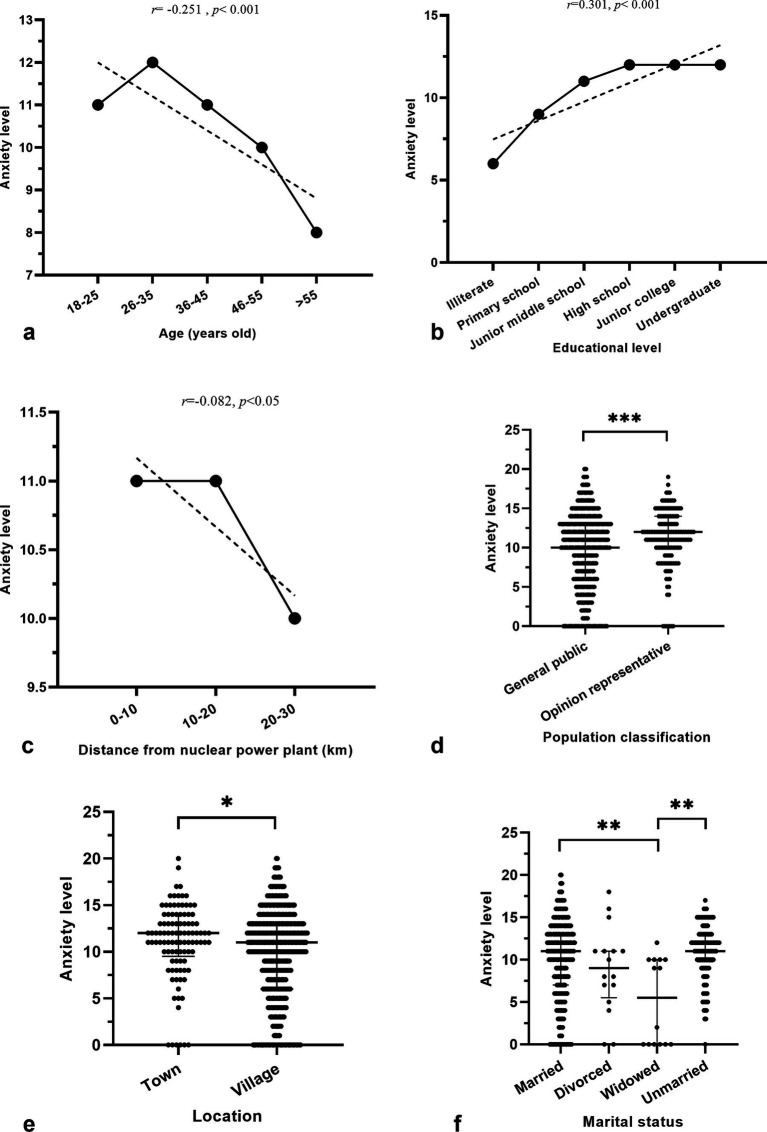
Factors influencing the anxiety level of respondents. **(A)** The influence of age on the anxiety level; **(B)** the influence of the educational level on the anxiety level; **(C)** the influence of the distance between residence and nuclear power plant on the anxiety level; **(D)** the influence of the population classification among the respondents on the anxiety level; **(E)** the influence of the location on the anxiety level; **(F)** the influence of the marital status on the anxiety level. The solid line represents the average anxiety level of the groups. *, *p* < 0.05. **, *p* < 0.01. ***, *p* < 0.001.

Spearman correlation analysis revealed significant associations between respondent anxiety levels and various demographic factors. Anxiety was inversely related to age, with older respondents exhibiting lower anxiety (*r* = −0.251, *p* < 0.001; Figure 2A). Conversely, anxiety was positively associated with education level, indicating that more educated respondents reported higher anxiety (*r* = 0.301, *p* < 0.001; Figure 2B). Additionally, anxiety was negatively correlated with the proximity of residence to the nuclear power plant, albeit with a weaker effect (*r* = −0.082, *p* < 0.05), suggesting that distance may mitigate anxiety(Figure 2C). When comparing population groups, the opinion representative group experienced greater anxiety than the general public (*p* < 0.001; Figure 2D). Urban residents also showed higher anxiety levels compared to those in rural areas (*p* < 0.05; Figure 2E). Furthermore, the marital status affected anxiety, with married and unmarried individuals reporting higher levels than the widowed group (*p* < 0.05; Figure 2F).

### 3.6 Multiple linear regression analysis of other influencing factors

Multiple linear regressions were conducted, considering population classification, sex, age, occupation, education level, distance from the nuclear power plant, location, whether there were family members working in the nuclear power plant, and marital status as independent variables as predictors (Table 4). Objective cognition levels of RRK and NERK, along with anxiety, served as outcome measures. The results showed that the RRK awareness level, NERK awareness and anxiety level of the residents around the nuclear power plant were positively correlated with the education level and negatively correlated with age (*p* < 0.05). In addition, NERK awareness level was negatively correlated with gender (*p* < 0.05), while anxiety level was negatively correlated with occupation and marital status (*p* < 0.05).

**Table 4:** Multiple linear regression analysis of residents’ RRK, NERK and anxiety level.

Variable	*b*	*S*_b_	*β*	*t* value	*p* value	95%*CI*
RRK						
Constant term	0.933	0.859	—	1.086	0.278	−0.753 ~ 2.620
Population classification	0.081	0.263	0.015	0.308	0.758	−0.435 ~ 0.597
Sex	−0.038	0.125	−0.009	−0.300	0.764	−0.283 ~ 0.208
Age	−0.297	0.068	−0.176	−4.384	**0.000**	−0.430 ~ −0.164
Occupation	−0.008	0.047	−0.007	−0.169	0.866	−0.100 ~ 0.084
Educational level	0.814	0.069	0.525	11.836	**0.000**	0.679 ~ 0.949
Distance from nuclear power plant	0.034	0.075	0.013	0.459	0.646	−0.113 ~ 0.182
Location	−0.125	0.203	−0.019	−0.616	0.538	−0.524 ~ 0.274
Family members	0.078	0.242	0.009	0.323	0.747	−0.396 ~ 0.552
Marital status	0.016	0.063	0.008	0.252	0.801	−0.108 ~ 0.139
NERK						
Constant term	1.225	0.791	—	1.549	0.122	−0.328 ~ 2.778
Population classification	−0.077	0.242	−0.017	−0.320	0.749	−0.552 ~ 0.398
Sex	−0.735	0.115	−0.191	−6.377	**0.000**	−0.962 ~ −0.509
Age	−0.134	0.062	−0.092	−2.154	**0.032**	−0.257 ~ −0.012
Occupation	−0.052	0.043	−0.055	−1.201	0.230	−0.137 ~ 0.033
Educational level	0.692	0.063	0.514	10.925	**0.000**	0.567 ~ 0.816
Distance from nuclear power plant	0.124	0.069	0.053	1.796	0.073	−0.012 ~ 0.259
Location	0.122	0.187	0.022	0.652	0.514	−0.245 ~ 0.490
Family members	−0.050	0.222	−0.007	−0.226	0.821	−0.487 ~ 0.386
Marital status	0.000	0.058	0.000	−0.005	0.996	−0.114 ~ 0.113
Anxiety level						
Constant term	12.265	2.340	—	5.241	0.000	7.671 ~ 16.859
Population classification	−1.306	0.715	−0.113	−1.828	0.068	−2.709 ~ 0.097
Sex	−0.109	0.341	−0.011	−0.319	0.750	−0.777 ~ 0.56
Age	−0.589	0.184	−0.163	−3.200	**0.001**	−0.95 ~ −0.227
Occupation	−0.263	0.127	−0.114	−2.060	**0.040**	−0.513 ~ −0.012
Educational level	0.893	0.187	0.269	4.782	**0.000**	0.526 ~ 1.259
Distance from nuclear power plant	−0.311	0.204	−0.053	−1.527	0.127	−0.712 ~ 0.089
Location	0.378	0.555	0.027	0.680	0.497	−0.713 ~ 1.468
Family members	0.000	0.656	0.000	0.000	1.000	−1.287 ~ 1.288
Marital status	−0.400	0.171	−0.094	−2.345	**0.019**	−0.735 ~ −0.065

The values in bold in the table represent *p* < 0.05, indicating statistical significance.

### 3.7 Other questions

#### 3.7.1 Confidence in the safety of nuclear power plants

**Figure 3:**
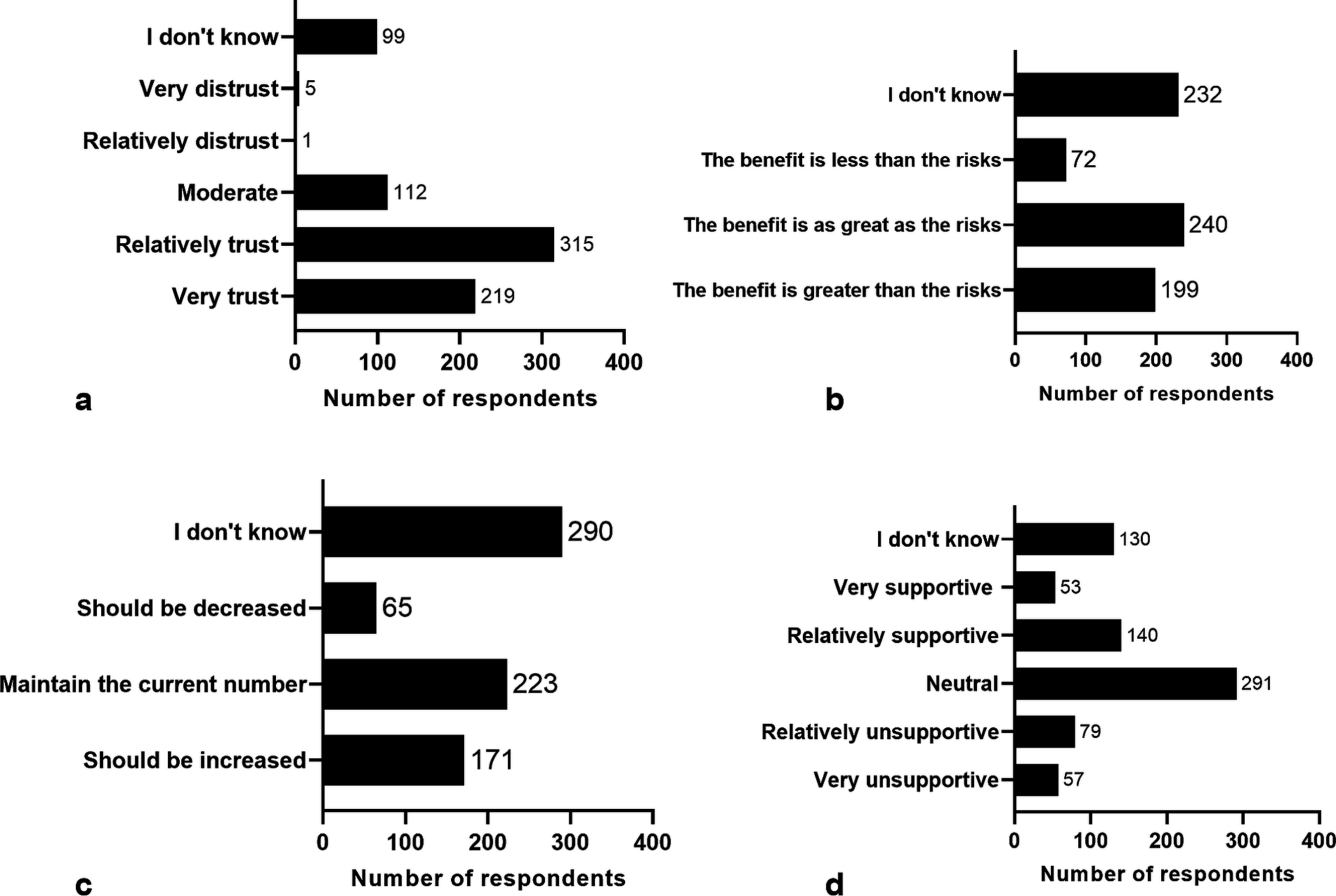
Statistics on the answers to questions in the questionnaire. **(A)** degree of confidence in the safety of our nuclear power plants; **(B)** thoughts on the benefits and risks of nuclear power; **(C)** thoughts on the construction of nuclear power plants in our country; **(D)** attitudes towards the construction of a nuclear power plant in the area where you live/work.

Survey results indicated that 71.1% (534 respondents) had strong or relatively strong trust in the safety of China’s nuclear power plants. Additionally, 14.9% (112) expressed a medium level of trust, while 0.8% (6) reported a relatively strong or very strong mistrust (Figure 3A).

#### 3.7.2 Perceptions of the benefits and risks of nuclear power plants

Considering both the benefits and risks of nuclear power, 32.3% (240 respondents) felt the benefits equaled the risks. This was followed by 31.2% (232) who were uncertain, 26.8% (199) who believed the benefits outweighed the risks, and 9.7% (72) who felt the benefits were less than the risks (Figure 3B).

#### 3.7.3 Perspectives on the construction of nuclear power plants in our country

Regarding the construction of nuclear power plants in China, 38.7% (290 respondents) were unsure. This was followed by 29.8% (223) who believed the current number should be maintained, 22.8% (171) who advocated for an increase, and 8.7% (65) who thought the number should be reduced (Figure 3C).

#### 3.7.4 Perceptions of the construction of nuclear power plants in residential areas

In the survey regarding the construction of nuclear power plants in respondents’ living or working areas, 25.7% (193 respondents) expressed very strong or relatively strong support, 38.8% (291) remained neutral, 18.1% (136) were relatively unsupportive or very unsupportive, and 17.3% (130) were unsure (Figure 3D).

Among those who feel the benefits of nuclear power outweigh the risks (26.5%, or 199 respondents), 45.7% (91) support increasing the construction of nuclear plants, 30.2% (60) want to maintain the current level, 4.5% (9) believe it should be reduced, and 19.6% (39) were uncertain.

Of the 22.8% (171 respondents) advocating for an increase in construction, within this group, 14.0% (24) were somewhat or very unsupportive of local nuclear plant construction, 30.4% (52) were neutral, 52.0% (89) were somewhat or very supportive, and 3.5% (6) were unsure.

### 3.8 Comparison of attitudes towards nuclear power plants in Zhejiang

**Figure 4:**
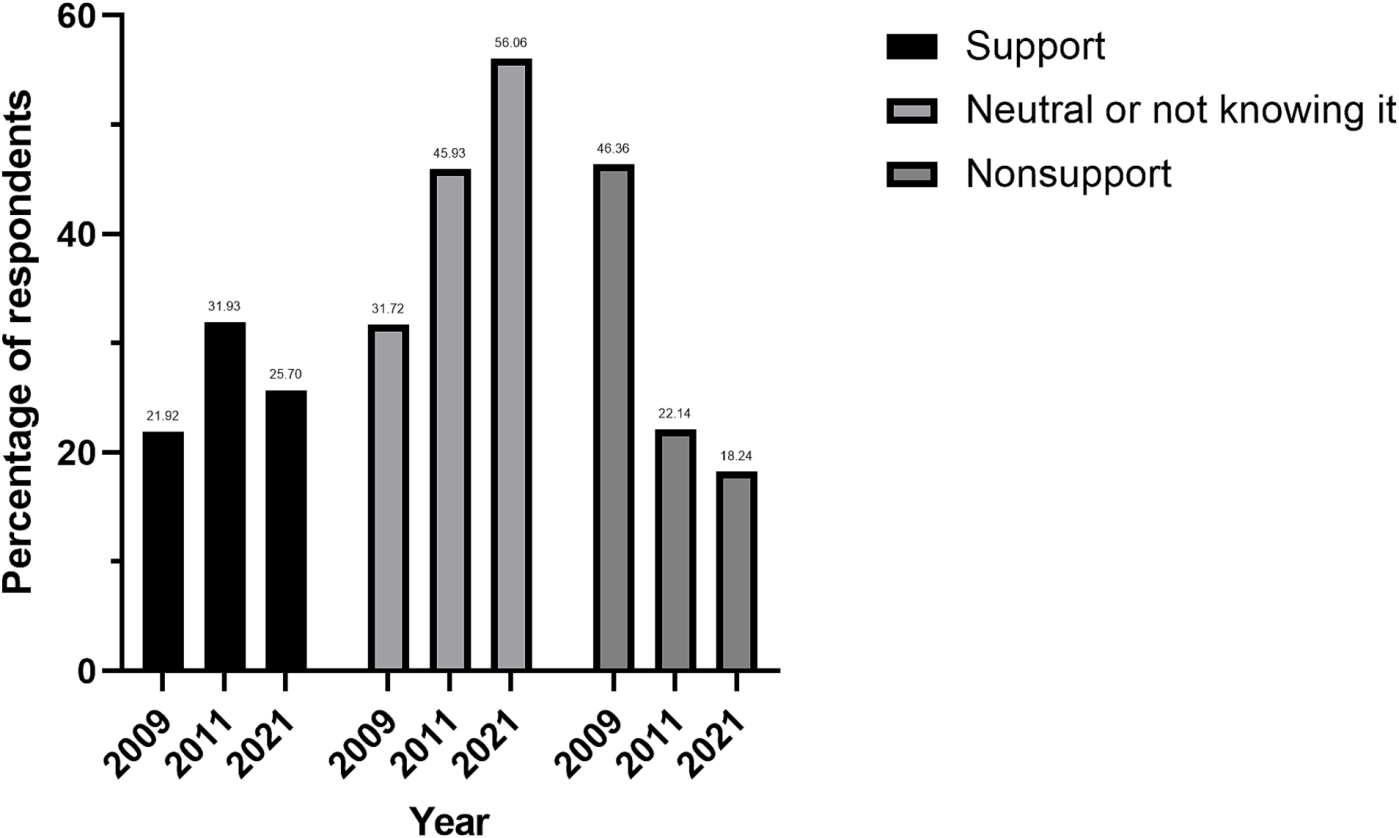
Residents’ attitudes towards nuclear power plants in successive surveys.

This survey’s findings build upon previous assessments, including the 2009 nuclear energy awareness survey conducted around the Qinshan Nuclear Power Station and the evaluation of cognitive perceptions of nuclear power among residents in the vicinity of the Sanmen Nuclear Power Station. All surveys gauged local sentiments on nuclear power plant construction. Support levels were highest in the 2011 Sanmen survey at 31.93%, followed by the 2021 Sanmen survey at 25.70%, and lowest in the 2009 Qinshan survey at 21.92%. Neutral or “do not know” responses increased over time, from 31.72% in 2009 to 56.06% in 2021. Conversely, opposition decreased, from 46.36% in 2009 to 18.24% in 2021. For detailed trends, refer to Figure 4.

## 4 Discussion

Nuclear power, with its low-carbon emissions, is pivotal for the global energy sector’s shift towards sustainability. Despite its proven necessity in energy revolutions worldwide, nuclear accidents have heightened public apprehension towards nuclear facilities, fostering opposition to nuclear development and use. This study surveyed 751 local residents, aged over 18 and within a 30-kilometer radius of the Sanmen Nuclear Power Plant, to assess their cognition of RRK and NERK, as well as their perspectives on nuclear plant construction. The sample size involved in this study exceeds 634 people (the calculated minimum sample size), thus the survey results were representative and could reflect the overall situation of the local residents.

The study revealed that residents near the Sanmen Nuclear Power Plant have a RRK cognition rate of 39.4%, similar to that of the Qinshan Nuclear Power Plant (39.6%) (14) and a proposed plant in Henan (39.5%) (15), but exceeded awareness in Sanmen’s immediate vicinity (34.4%) (16)and adjacent areas (32.3%) (17). It fell short, however, when compared to the Tianwan Nuclear Power Plant (56.7%) (18). NERK awareness among Sanmen residents standed at 30.5%, aligning with the Henan proposal (29.7%), surpassing Sanmen’s adjacent regions (12.9%), and lagging behind Tianwan (56.7%). The data indicated a modest increase in awareness from previous years, suggesting that broader integration and education on nuclear energy have improved residents’ understanding, albeit from a low base. Discrepancies between subjective and objective awareness evaluations highlighted the importance of objective measures, correlating these evaluations with educational levels and suggesting greater understanding with higher education. Opinion representatives exhibited higher awareness, potentially due to advanced education. Spearman correlation analysis and multiple linear regression analysis indicated that education level and younger age were positively associated with RRK and NERK awareness, with the latter also revealing a gender impact, suggesting females have a lower level of understanding than males. The study showed that younger individuals had higher awareness of radiation and nuclear energy, likely due to modern education in science and technology (19). Older individuals, lacking such exposure, exhibited lower awareness, compounded by age-related cognitive decline. Education level significantly impacted health literacy and scientific understanding, with higher education correlating to better comprehension of complex topics (20). The impact of gender on the level of NERK may stem from the fact that males, due to societal roles and interests, are more inclined to engage with topics related to technology, engineering, and science (14, 19). In comparison, radiation is more related to health and environmental issues, which may not differ much in attention between genders, resulting in no significant gender differences in NERK.

The study’s findings revealed an inverse relationship between respondents’ anxiety levels and age, coupled with a direct correlation with education level, mirroring the observed awareness of RRK and NERK. This trend likely stemmed from older individuals’ limited education due to lower national education rates during their upbringing. Conversely, higher education levels were associated with a deeper understanding of nuclear power plant risks, potentially increasing anxiety. Anxiety levels also showed a negative correlation with proximity to nuclear power plants, indicating that those living at a futher distance have reduced concerns about nuclear safety. This aligned with research suggesting that residents in close proximity to nuclear facilities harbored higher anxiety levels due to perceived accident risks. Representatives of opinion groups and urban dwellers reported higher anxiety levels than the general public and rural residents, respectively. This might be due to their increased exposure to negative information regarding nuclear power plants. Furthermore, married and unmarried individuals exhibited significantly higher anxiety than those who were widowed, possibly linked to the familial and life pressures of those in or entering marriage. Regression analysis substantiated the influence of age, education, occupation and marital status on nuclear power-related anxiety. Accordingly, it is recommended that authorities enhance radiation and nuclear energy education for residents in the vicinity of nuclear power plants to mitigate anxiety.

The survey also highlighted varying opinions on the benefits and risks of nuclear power plants, as well as attitudes towards their construction in residential and work areas. A significant 26.8% believe nuclear power’s benefits surpassed its risks, potentially due to the economic growth these plants can bring to local areas. In contrast, 9.7% believed the risks were greater, likely due to fears of nuclear accidents, indicating a disparity in public understanding of nuclear energy’s pros and cons. Moreover, 22.8% were in favor of increasing the number of nuclear power plants, while 8.7% opposed it. This split may stem from differing views on energy demands, environmental conservation, and economic progress. Proponents likely appreciated the economic advantages and nuclear energy’s role in reducing greenhouse gas emissions, while opponents might prioritize safety hazards and the long-term ecological consequences. Additionally, 25.7% supported the construction of nuclear power plants in their local areas, while 18.1% were against it. Support may be driven by expectations of job creation, economic boost, and clean energy supply, while opposition could be rooted in concerns over health, environmental effects, and the impact on living standards (21).

The 2009 survey around the Qinshan Nuclear Power Station indicated that 21.92% of respondents were in favor of, or more supportive of, local nuclear power plant construction, a figure that rose to 31.93% in the 2011 Sanmen Nuclear Power Station survey. By 2021, this figure experienced a slight decline to 25.70%. These fluctuations suggest that while the Fukushima nuclear accident initially dampened public awareness of nuclear power, a consistent subset of the population remains supportive of nuclear energy initiatives over time (14). From 2009 to 2021, there was a notable increase in the proportion of respondents who were neutral or unaware, climbing from 31.72 to 56.06%. This trend may indicate growing public uncertainty or a heightened demand for information regarding nuclear power stations. Conversely, the percentage of those opposed to, or not in favor of, nuclear power plant construction saw a significant drop from 46.36% in 2009 to 18.24% in 2021. This decline could signify a growing public understanding and acceptance of the safety and necessity of nuclear power plants. Despite initial concerns post-Fukushima, public attitudes have gradually shifted as information dissemination and scientific literacy have improved. This underscores the importance of ongoing communication and education in shaping public perception of nuclear power (15). Public sentiment is a pivotal factor in the advancement of nuclear power, significantly influencing policy development, technological progress, and economic feasibility. Enhancing public acceptance of nuclear energy is essential and can be achieved by bolstering educational outreach on nuclear science, encouraging public engagement, and fostering trust between the community, nuclear energy corporations, and governmental bodies. Effective communication and transparency are instrumental in providing the public with a more holistic understanding of the benefits and risks associated with nuclear power, enabling them to make more informed decisions.

## 5 Conclusion

This study conducted a comprehensive analysis of the cognition of RRK and NERK, anxiety levels, and attitudes towards nuclear power plant construction among residents. While residents near the Sanmen Nuclear Power Station, the first project adopted the AP1000 technology, had shown improvements in RRK and NERK, their levels remained relatively low. Both RRK and NERK correlated with age and education level, with NERK additionally linked to gender. Anxiety levels among residents were associated with age, education, occupation and marital status. Furthermore, support and opposition to nuclear power plant construction had diminished among residents near the Sanmen Nuclear Power Station, with an increasing number expressing ambiguity and uncertainty. These findings underscore the need for enhanced public education on RRK and NERK. To boost RRK and NERK awareness among diverse age groups and education levels, especially the older adults and less educated, targeted education programs are essential. Community meetings and public lectures can enhance understanding and trust in nuclear facilities, dispelling fears and myths. Integrating nuclear safety into school curricula near nuclear plants fosters early scientific literacy. Broadcasting scientific knowledge via TV, radio, newspapers, social media, and printed materials is crucial. Gender-sensitive strategies ensure equal knowledge access for all genders. Regular assessments of educational and training programs are necessary to ensure their effectiveness and to make adjustments based on feedback. These systematic efforts will improve residents’ awareness and cultivate a culture of nuclear safety. Concurrently, it is imperative for the government and relevant departments to focus on effective public engagement and negotiation to improve decision-making quality, bolster social trust, and elevate the overall safety of nuclear power.

## Data Availability

The original contributions presented in the study are included in the article/Supplementary material, further inquiries can be directed to the corresponding authors.
